# Low-Dose Ionizing Radiation and Myalgic Encephalomyelitis/Chronic Fatigue Syndrome (ME/CFS): A Review of Recent Evidence and Future Research Directions Toward the Elucidation of a Metabolic, Immunologic, and Signaling Cascade

**DOI:** 10.3390/ijms27146535

**Published:** 2026-07-22

**Authors:** Andrej Rusin, Alan Cocchetto, Carmel Mothersill

**Affiliations:** 1 Department of Integrated Biomedical Engineering and Health Sciences, McMaster University, Hamilton, ON L8S 4L8, Canada; rusina@mcmaster.ca; 2 National CFIDS Foundation Inc., Hull, MA 02045, USA; acocchetto@gmail.com; 3 Department of Biology, McMaster University, Hamilton, ON L8S 4L8, Canada

**Keywords:** radiation-induced bystander effect (RIBE), low-dose ionizing radiation, mitochondria

## Abstract

Myalgic encephalomyelitis/chronic fatigue syndrome (ME/CFS) is an idiopathic, multisystem disorder marked by debilitating fatigue, post-exertional malaise, cognitive dysfunction and neuroinflammation. Its etiology remains unclear, yet emerging evidence implicates a complex interplay between immune dysregulation, metabolic impairment, mitochondrial bioenergetics, and environmental stressors such as viral infection or low-dose ionizing radiation (LDIR). To develop treatments for ME/CFS, it is essential to identify suitable targets for therapy. In this narrative review, we discuss recent findings on the overlap of ME/CFS with LDIR effects and examine potential mechanistic links that may arise from LDIR-induced bystander effects (RIBEs). We highlight potential candidate biomarkers that bridge these domains: mitochondrial respiratory dysfunction, altered ornithine transport via SLC25A15 (ORNT1), possible roles of CD38 in the context of immunity and NAD+ depletion, cyclin D1–dependent metabolic reprogramming and modulation of gene expression, and α-synuclein as a potential neuroinflammatory damage-associated molecular pattern (DAMP). While the involvement of these biomarkers in ME/CFS is yet to be confirmed experimentally, evidence from in vitro studies of irradiated cells, exosome profiling, and patient samples suggests that RIBEs can, in theory, produce prominent cellular ME/CFS phenotypes through associated mechanisms, including those exhibiting oxidative stress, impaired ATP production, and immune modulation. We propose a hypothetical, exploratory model wherein LDIR initiates or contributes to adaptive metabolic shifts (including CD38 upregulation and cyclin D1 stabilization) that, coupled with persistent bystander signaling, could potentially culminate in chronic fatigue and neurocognitive symptoms in some reported ME/CFS cases. Finally, we outline a research agenda encompassing the establishment of standardized diagnostic criteria, multi-omics profiling of patient cohorts, exosome analysis, functional mitochondrial assays, and targeted therapeutic trials focusing on possible anti-CD38 antibodies and NAD+ precursor therapy. By integrating recent findings in low-dose radiation biology with ME/CFS pathophysiology, this review aims to promote interdisciplinary investigations that may uncover mechanistic insights and novel biomarkers for diagnosis and treatment of ME/CFS. We further review steps in a proposed model taking us from low-dose radiation exposure to a number of possible targets.

## 1. Introduction to Myalgic Encephalomyelitis/Chronic Fatigue Syndrome (ME/CFS)

Chronic Fatigue Syndrome, also known as Myalgic Encephalomyelitis/CFS (ME/CFS) and Chronic Fatigue and Immune Dysfunction Syndrome (CFIDS), is a debilitating condition that severely impairs the ability of patients to go about their daily lives [1,2,3]. Patients are typically unable to engage in activities that were tolerated previously due to persistent fatigue and associated symptoms that cannot be ameliorated through rest [4]. Other common symptoms of ME/CFS include persistent cognitive difficulties, post-exertional malaise, vertigo, muscle pain, tender lymph nodes, and unrestful sleep [5]. A diagnosis of ME/CFS is usually made following exclusion of other diseases, and the diagnosis utilizes one of several sets of criteria [6]. In the United States, the Fukuda Criteria for ME/CFS diagnosis were applied historically following a differential diagnosis, which emphasized clinically evaluated yet unexplained, persistent, and relapsing fatigue that could not be alleviated through rest, along with several secondary symptomatic criteria [1]. Several other proposed diagnostic criteria are employed in clinical practice today with more exclusionary definitions, including the Canadian Consensus Criteria (CCC) and the (US) Institute of Medicine Criteria (IOM), which identify post-exertional malaise (PEM) as a cardinal symptom [7,8]. Though the revised CCC may enhance specificity and afford more homogeneity among patient groups in studies investigating ME/CFS etiology, the CCC excludes patients who do not meet its strict criteria and, therefore, may not capture a full “spectrum” of ME/CFS cases; this may unintentionally prevent some patients suffering from debilitating fatigue from being thoroughly evaluated and treated [8,9]. The diversity of diagnostic criteria and lack of a universally accepted set of criteria make it difficult to compare and glean new information from different studies on ME/CFS; since different studies oftentimes utilize different diagnostic criteria and definitions, patient and control groups may vary considerably between publications [3]. For example, many researchers and patients contend that PEM is a hallmark symptom of ME/CFS, which often distinguishes the disease from possibly related syndromes like Idiopathic Chronic Fatigue (ICF) and Post-Acute Infection Syndrome (PAIS) [10,11].

Though the disease affects millions globally, and many groups have investigated its progression and etiology, the underlying cause or causes remain elusive [12]. Several authors have proposed that ME/CFS may even be a variety of related conditions that can be categorized into subgroups, with post-exertional malaise being a ubiquitous symptom [13,14,15,16,17,18,19]. Presently, the literature is still contentious but establishes a strong case across research groups that ME/CFS is a physiological condition and likely involves a complex interplay of metabolism, immunity, and neuropathy in the emergence and progression of the disease [20,21,22,23,24,25].

Many ME/CFS cases begin after acute infection or after exposure to a stressor; the observed preceding infections in ME/CFS are typically viral [26,27]. In fact, many of the symptoms which constitute some of the commonly accepted diagnostic criteria overlap considerably with PAIS [10,11]. There has been considerable research into whether infection by a specific virus directly causes ME/CFS; however, clear etiological connections between any infection and ME/CFS manifestation have not been definitively established [11,26,27]. For example, a significant percentage of individuals suffering from Long COVID, a protracted type of PAIS that occurs in some following recovery from acute COVID-19, exhibit symptoms that overlap with ME/CFS, depending on the diagnostic criteria being applied [22,28,29]. Further, a significant percentage of patients with infectious mononucleosis, a disease primarily caused by the Epstein–Barr virus, develop symptoms that meet the diagnostic criteria of both PAIS and ME/CFS [26,30,31,32,33,34,35,36]. Other known viruses that may contribute to the development of ME/CFS in some cases include SARS-CoV-1 [5,37], varicella zoster virus [38], and influenza virus A subtype H1N1 [39,40]. It is important to note that other stressors have been identified as potentially causative in other ME/CFS cases, including bacterial infections and disrupted gut microbiota [41,42,43], psychological stress [44,45,46], traumatic injury [47,48], pregnancy [49], and exposure to mold [50,51,52].

Beyond infection, autoimmune models propose that infectious or other environmental stressors may promote sustained immune dysregulation in ME/CFS, leading to the production of autoantibodies against adrenergic or muscarinic receptors, which could possibly contribute to observed symptoms such as orthostatic intolerance, fatigue, and cognitive impairment [53,54,55]. Additional findings in this area, including altered B-cell function, T-cell abnormalities, and associations with specific human leukocyte antigen (HLA) alleles in some cohorts, further support the possibility of autoimmune involvement in ME/CFS [55,56,57,58]. These findings have not been consistently replicated across all patient populations, indicating that autoimmunity may likely be relevant only in a subset of individuals with ME/CFS.

Additionally, neuroinflammatory models propose that persistent activation of microglia and astrocytes within the central nervous system leads to chronic production of pro-inflammatory cytokines and other inflammatory mediators in ME/CFS [59,60,61]. This sustained neuroimmune activation may disrupt neuronal signaling, alter neurotransmitter homeostasis, and impair autonomic regulation. This has been proposed to result in fatigue, cognitive dysfunction (or “brain fog”), sleep disturbances, and post-exertional malaise. Peripheral immune activation, mitochondrial dysfunction, oxidative stress, and blood–brain barrier alterations may further amplify these neuroinflammatory processes. Although direct evidence of neuroinflammation in ME/CFS remains mixed due to methodological differences in studies and disease heterogeneity, accumulating neuroimaging, cerebrospinal fluid, and immunological studies support the hypothesis that neuroimmune dysfunction is an important component of disease pathogenesis.

In this report, we describe how low-dose ionizing radiation (LDIR) exposure could potentially be linked to ME/CFS. We propose that our hypothetical LDIR model should be considered complementary to, rather than competitive with, existing hypotheses of ME/CFS pathogenesis. Rather than replacing infectious, autoimmune, or neuroinflammatory models, LDIR exposure may represent an upstream environmental trigger capable of initiating or amplifying the biological processes implicated in these established mechanisms. Radiation-induced oxidative stress, persistent immune activation, mitochondrial dysfunction, and neuroinflammation, as described in this review, could potentially contribute to the development of persistent immune dysregulation, microglial activation, and autonomic dysfunction that characterize ME/CFS. Within this framework, we propose that LDIR is not a universal cause of the disease but rather one of several potential initiating factors that may contribute to disease onset in susceptible individuals, ultimately converging on shared downstream pathways responsible for the clinical manifestations of ME/CFS.

### Atomic Veterans and Connections to Low-Dose Ionizing Radiation Exposure

Many individuals exposed to ionizing radiation, following nuclear catastrophes (e.g., Chernobyl, Fukushima), the deliberate use of nuclear weapons in war (e.g., Hiroshima, Nagasaki), and inadvertent exposure following nuclear weapons tests (e.g., Marshall Islands, other Cold War tests), have experienced diverse and long-lasting health effects that overlap with the symptomatology of ME/CFS [62,63,64,65,66,67,68,69,70,71,72,73,74]. While the primary focus of radiation protection efforts and studies has been, for decades, to mitigate the emergence of excess cancers due to high-dose exposures, much less focus has been afforded to the other effects of Post-Radiation Syndrome (PRS) that may occur even at low doses [75,76]. Many of those directly affected by these radiation events assert that their symptoms could be the result of ionizing radiation exposure even at lower doses; however, much of the time, they are met with the obstinate incredulity of clinicians and officials who do not believe that their symptoms can be causally linked to radiation exposure. The basis of this belief relies on two ideas: that low doses cannot produce such health effects, especially many years after the initial exposure, and that there are insufficient data to conduct epidemiological analyses to form a causal link between exposure and disease [75]. Based on decades of research into low doses and the non-targeted effects of ionizing radiation, both premises can no longer be justified by evidence [77,78,79,80,81,82,83]. In brief, the commonly applied linear no-threshold model (LNT), which is often used inappropriately to exclude the involvement of low doses in the etiology of disease beyond stochastic cancer risk, is no longer sufficient in modeling the effects of radiation exposure in the realm of low-dose (<100 mSv) effects. The effects of low doses of radiation on organisms are highly context-dependent (i.e., radiosensitivity of the organism, type of radiation used, tissue(s) exposed, radiation energy, fractionation, et cetera) and can involve persistent transgenerational effects [84,85], DNA damage [86,87,88], complex bystander signaling cascades [89,90,91,92], altered metabolism [93,94,95], inflammatory/immune responses [91,96], oxidative and nitrosative stress [86,87,93,94,95,97,98,99], and other effects that cannot reliably be predicted by standard models of stochastic risk which rely on historical data from high-dose exposures and records of excess cancers [75]. The merits and drawbacks of LNT will not be extensively discussed in this review, but we have extensively covered the subject in previous reviews. For further reading on LNT, please see [78,82,83,100,101,102].

**Figure 1:**
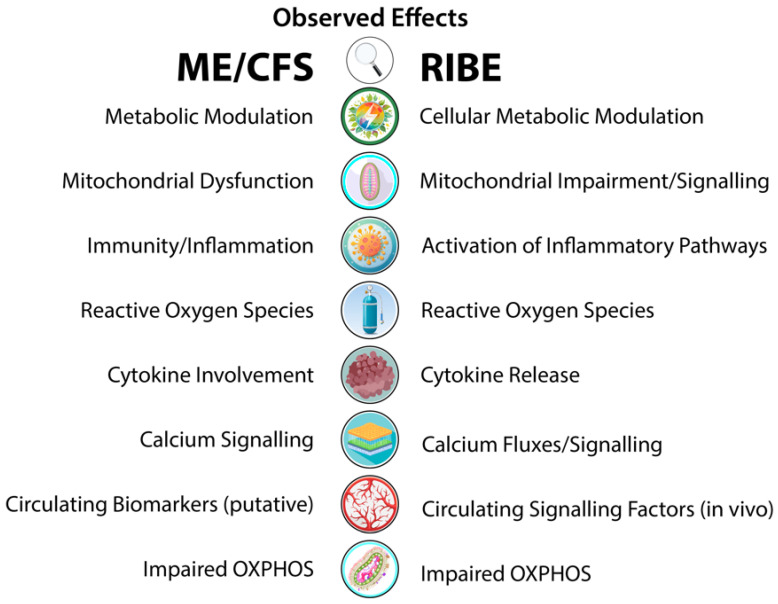
A diagram illustrating the overlap in observed effects between ME/CFS and RIBEs that are discussed in this review.

The primary interest in ME/CFS and how it may be connected to radiation exposure began following publication of reports in the late 1990s and early aughts that concerned clinical assessments of cohorts of Chernobyl liquidation workers. These reports found symptoms consistent with ME/CFS, including persistent fatigue, cognitive issues, sleep disturbances, and mood changes even years following exposure [62,63,103,104,105,106]. Important to note are several limiting aspects of these studies in order to provide some context to these findings. While the studies did report significant symptoms in a large proportion of the liquidators, even at doses below 300 mSv, it is fair to argue that the small sample size of approximately 100 liquidators used in the initial study should be broadened to strengthen its conclusions. Additional research is certainly required in this area, and we advocate for large-scale, well-controlled epidemiological studies to test these conclusions. However, it is interesting to note these effects even considering small sample sizes because there does not currently exist a databank of biological samples representative of these liquidators or any other patients afflicted with ME/CFS that can be further scrutinized. We outline several potential avenues of research within this narrative review that could assist in determining what potential biomarkers could be used to identify LDIR exposure and what possible related mechanisms could be relevant to ME/CFS pathogenesis. A second potential issue with these studies is that the liquidators faced not only radiation exposure but also additional stressors, such as significant psychological trauma, social disruption, socioeconomic distress, exposure to heavy metals, and extreme working conditions, which could have contributed to ME/CFS symptoms. We conversely believe this further implies that ME/CFS pathogenesis is likely related to exposure to a combination of stressors that promote the manifestation and maintenance of ME/CFS symptoms and that any new research endeavoring to help elucidate the mechanisms by which this occurs would be optimal to pursue. Additionally, another ostensible problem with these studies is the lack of a clear dose-dependent relationship between radiation dose and symptoms. However, we contend that this could be further evidence that low-dose effects are not clearly dose-dependent in all circumstances, especially given the presence of additional stressors that could modulate any biologically adaptive or maladaptive responses to the radiation. Collaborative research within our group has found that radiation-induced bystander effects (RIBEs) can be induced in immortalized keratinocyte reporters by sera from survivors of the Chernobyl Disaster, including liquidator cohorts, some twenty years following the accident [107]; further investigation is required to elucidate these effects further and probe for relevance in the context of diseases such as ME/CFS. Figure 1 shows some of the observed effects and similarities between ME/CFS and RIBE.

There is considerable overlap with the symptoms of PRS at first glance. In high-dose exposures, early symptoms include headache, dizziness, weakness, fatigue, anorexia, and nausea and vomiting [108]. In lower- or targeted-dose exposures, which are more pertinent to radiotherapy, extreme fatigue is a common symptom. In fact, delayed fatigue in those undergoing external beam radiation therapy (EBRT) is both a common and extensively researched symptom in the context of cancer therapy [109,110,111,112]. Somnolescence Syndrome occurs in some as a delayed response to radiation treatment, most commonly in the treatment of brain tumors [113,114]. This syndrome is characterized by extreme fatigue, malaise, loss of appetite, and vertigo.

Again, it is important to recognize that many diseases cause fatigue and associated symptoms. We suggest that a broad examination of potential factors that cause these symptoms may result in a more robust understanding of the underlying pathophysiology in ME/CFS and syndromes associated with low-dose radiation exposure. There are some very interesting potential links between ME/CFS and LDIR exposure that will be discussed in this review. However, these putative connections and mechanistic links still require extensive in vitro and clinical investigation to validate before firm conclusions can be made.

## 2. Metabolism and Signaling in ME/CFS

### 2.1. Hypometabolism and Current Research Limitations

There has been extensive research into reliable biomarkers for ME/CFS, with limited success at achieving consensus on standard biochemical testing and diagnosis [115,116]. As discussed previously, major problems exist in contemporary research due to small sample sizes, varied endpoints, disparate samples from different tissues, and inconsistent diagnostic criteria. A recent systematic review [117] covered studies on a range of potential biomarkers in several broad categories, including genetic, metabolic, circulatory, neurological, ion channel, and “physical dysfunction”. While it was found that several studies validated the use of lymphocytes as a model to investigate etiology and validated evidence of immune dysfunction, the overall reproducibility of these studies was limited. The authors suggested the establishment of uniform protocols and the need for multidisciplinary research to address the heterogeneity of findings in future research.

There is considerable evidence that hypometabolism or otherwise disrupted metabolism in various tissues plays a major role in ME/CFS, and that this dysfunction likely represents a complex interplay between immunity, metabolism, neuropathology, and inflammation [117,118,119,120,121,122,123]. We will examine a few cellular processes and metabolic pathways that have been investigated in ME/CFS literature and several with suspected yet currently unconfirmed involvement. A recurring theme in this research is the close examination of mitochondria due to their close involvement in many different metabolic pathways and cellular processes [124,125]. We believe that these mechanisms may potentially link specific ME/CFS cases to LDIR exposure, but further research must be conducted before any firm conclusions can be drawn.

### 2.2. The Role of Mitochondria in ME/CFS

Mitochondria are well known as the primary sites of cellular energy production, generating adenosine triphosphate (ATP) through the mitochondrial respiratory chain and specifically through oxidative phosphorylation (OXPHOS) [126]. Mitochondria are also essential in many metabolic processes including pyruvate oxidation, pyruvate carboxylation, the citric acid cycle, and fatty acid synthesis [126]. Further, mitochondria are crucial in signal transduction in the cell, facilitating or participating in calcium signaling, programmed cell death, cellular proliferation, innate immunity, and hormonal signaling [127]. As a nexus of cellular metabolism and energy production, many researchers have long hypothesized that perturbations of mitochondrial bioenergetics may contribute to the emergence and severity of symptoms in ME/CFS [124,125]. It appears likely based on current literature that mitochondrial function is implicated in ME/CFS etiology either as a secondary result of disease progression or as an underlying cause of the diseased state.

Many studies in the past have reported defects in mitochondria from ME/CFS patients [124,128,129,130,131,132]. These have suggested that overall mitochondrial function is potentially compromised in ME/CFS patients compared to healthy controls. A common, simple biomarker used to assess mitochondrial function is overall production of ATP through Complex V. Previous research has shown defects in OXPHOS pathways and reduced ATP production in ME/CFS patients, and that this reduction can correlate with clinical fatigue symptoms [129]. In a meta-analysis of data collected from work on the peripheral blood mononuclear cells (PBMCs)—which encompasses T cells, B cells, NK cells, and monocytes—from ME/CFS patients, researchers reported significantly lower basal respiration, maximal respiration, ATP production, proton leakage, and reserve respiratory capacity compared to healthy controls [130]. Further research has examined elevated lactate coincident with inadequate ATP production in blood cells from ME/CFS patients [130,133] and hypothesized that the mitochondrial pyruvate dehydrogenase complex may be impaired in some ME/CFS patients. Studies on PBMCs from ME/CFS patients [20,120,133,134,135] indeed demonstrated an impairment of the TCA cycle and protein catabolism, suggesting possible functional impairment of pyruvate dehydrogenase. Interestingly, one study found that myoblasts grown in the presence of serum from ME/CFS patients showed increased mitochondrial respiration and secretion of lactate [133], possibly indicating the presence of circulating signaling factors. However, these findings are yet to be replicated in larger-scale studies.

Recent studies of mitochondrial function in the skeletal muscle of ME/CFS patients continue to provide evidence of abnormalities compared to healthy controls. A study by Bizjak et al. [136] found evidence of impaired mitochondrial function in the muscle fibers from patients suffering from ME/CFS and post-COVID Syndrome (PCS), respectively, compared to healthy controls. Further, the same study found differences in distribution of the OXPHOS capacity of ME/CFS patients compared to healthy controls; however, the mean difference between the three groups was not statistically significant. A significant difference between the PCS and ME/CFS groups was revealed in electron micrographs evidencing “progressed pathological morphological changes in [ME/CFS mitochondria]”. The authors surmise that these structural changes to mitochondria may indicate “prolonged inactivity or unknown molecular causes” but strongly encouraged further investigation. Other studies [28,137] have proposed that mitochondrial calcium signaling may be connected to these changes, though further research must be done to find a conclusive link.

Another study on lymphoblast cell lines established from ME/CFS patients found significant reductions in ATP-synthase (Complex V) activity [138]. While compensatory glycolysis and mitochondrial mass remained the same under conditions of stress, significant upregulation of Complex I oxygen consumption, expression of respiratory enzymes, expression of fatty acid transporters, and TCA cycle enzymes was observed. The authors concluded that while enzymatic activity, expression, and ATP synthesis can return to steady state in a state of rest, the same cells may not be able to “adequately respond to acute increases in energy demand as the relevant homeostatic pathways are already activated” [138]. The same group expanded their findings in a later transcriptomic and proteomic study [139] on lymphoblast cell lines from ME/CFS patients and healthy controls by examining the pathways suspected to be dysregulated in ME/CFS. The authors reported similar regulation of glycolysis between the two groups but noted significant elevation in the enzymes involved in the TCA cycle, pentose phosphate pathway, mitochondrial fatty acid oxidation, glutamine/glutamate catabolism, branched chain amino acid catabolism, and the catabolism of essential fatty acids. The authors concluded that the compensatory metabolic activity in ME/CFS may involve processes that provide oxidizable substrates to mitochondria over glycolysis. Further, there is evidence to suggest both morphological and metabolic abnormalities in the mitochondria of other leukocytes, such as NK-leukocytes [131]. However, further research must be done to establish the involvement of these potential biomarkers in ME/CFS pathogenesis.

A very interesting clinical study examining the role of mitochondrial signaling and immunity in the context of ME/CFS [140] found that mitochondrial DNA (mtDNA) associated with serum exosomes is elevated in ME/CFS patients following exercise. Further, these isolated exosomes were found to promote significant release of IL-1β, a pro-inflammatory cytokine and mediator of inflammatory responses, from cultured human microglia in vitro. These findings suggest that the pathogenesis of ME/CFS likely involves a dynamic interplay of mitochondrial involvement, broader bioenergetics, immunity, and the central nervous system, and may even implicate mitochondria as direct effectors of extracellular signaling in ME/CFS pathophysiology. Exosomal signaling must first be justified by additional clinical studies before this conclusion is accepted.

Many groups have attempted to integrate these findings into a model for mitochondrial dysfunction in ME/CFS etiology. A common theme is excess oxidative stress [28,129], which has long been suspected to disrupt membrane potential and ATP synthesis in the mitochondria of patients. Further, it appears that there is now overwhelming evidence that metabolic dysfunction is present in the majority of ME/CFS cases [20,129,133,141,142,143], and that aberrant oxidative metabolism may precipitate compensatory shifts in mitochondrial metabolism to establish homeostasis, as hypothesized in previous studies. There appears to be considerable variance in data from ME/CFS patients compared to healthy controls across different studies, which may indicate that ME/CFS is a heterogeneous disease that could involve a myriad of elements and causes between different patients. More research is required to determine whether these phenomena can be possibly used to differentiate putative subtypes and/or severities of ME/CFS and if such alterations may cause progression of the disease or result due to the progression of the disease. If the latter is the case, it may possibly point towards mitochondrial dysfunction in ME/CFS being a consequence of systemic immune and inflammatory dysregulation, but these mechanisms are under active investigation. The findings in the current literature, though compelling, should be confirmed by studies using larger sample sizes to further elucidate common trends. It is apparent that limitations may also be addressed by promoting a set of common diagnostic criteria for ME/CFS and standardizing the replication of key results representative of mitochondrial dysfunction in relevant samples [133], such as assay of ATP-synthase activity. Additionally, many recent studies have incorporated testing ME/CFS patients during post-exertional recovery [23,144] and comparing these findings to healthy controls. This is a good direction for future research because metabolic abnormalities related to mitochondrial function are likely to manifest following a period of exertion and during recovery and during a period of time when ME/CFS symptoms are most likely to be experienced by patients [24,129,144,145].

### 2.3. Mitochondria in Low-Dose Radiation Research and RIBE

As previously discussed, mitochondria may provide a tentative link to low-dose radiation exposure in some patients with ME/CFS and similar fatigue syndromes. There is considerable evidence that RIBEs involve the release of exosomes from cells either exposed to ionizing radiation directly or having received bystander signals [146,147,148,149,150]. Previous studies have found that non-coding RNAs, including some miRNA and lncRNAs, may be involved in the transmission of the bystander signal to neighboring cells through exosomes in irradiated cell-conditioned medium (ICCM) [146,151]. These ICCM exosomes have also been found to contain mtDNA [147], which may be similar to the potential “pro-neuroinflammatory exosomes” harboring mtDNA in the sera of some ME/CFS patients [140]. However, further research is necessary to confirm these findings in a broader selection of patients and identify whether additional factors, such as ncRNAs, may also be implicated in ME/CFS signaling.

Mitochondria are central to both cellular metabolism and signaling, and their role in mediating RIBEs and likely ME/CFS is evident in the literature. Mitochondria and their associated metabolic pathways are incredibly complex and require the coordination of hundreds of different proteins and other biomolecules to function properly [126,127]. Obvious targets of future proteomic/transcriptomic/kinetics research endeavoring to link LDIR exposure and ME/CFS would be major enzymes and enzymatic complexes already known in ME/CFS and low-dose effects, such as the mitochondrial respiratory complexes, pyruvate dehydrogenase, TCA cycle enzymes, inner- and outer-membrane translocases, and others [23,92,124,125,128,132,136,138,147,152,153,154]. It is also probable, based on previously reviewed research on mitochondrial aberrations in ME/CFS and because these pathways are connected to larger metabolic and signaling networks, that mitochondria must be involved in the etiology of ME/CFS in some capacity.

### 2.4. Putative Biomarkers of Interest in ME/CFS and RIBE Research

The involvement of some mitochondrial proteins has already been clinically demonstrated in ME/CFS, as reviewed in previous sections. Additional metabolomic and proteomic research is required to establish standard testing practices and diagnostic criteria to then be investigated in the context of LDIR exposure as well. Such research could identify potential players affected by indirect radiation effects that could potentially be linked to ME/CFS in specific patients if common trends in metabolic impairment can be deduced. If specific proteins are identified as significantly different among patients and between studies utilizing the same methodology, we suspect that commonly affected enzymes in ME/CFS will be additionally involved in multiple cellular processes that overlap multiple metabolic pathways and may further be implicated in cell-to-cell signaling, immunity, and inflammation, as described in other, related models. A “holistic” model of these systems, one that incorporates features and key players identified in the ME/CFS literature, is almost certainly required to appreciate multiple ostensibly disparate biological processes that coordinate to manifest a diseased state. Furthermore, even if such a model provides a yet-incomplete understanding of the pathogenesis or maintenance of ME/CFS, such a model can nevertheless potentially inform future research endeavoring to elucidate these mechanisms further to a point where ME/CFS can be effectively prevented or even treated. Mitochondria, as previously described, are likely to be involved either directly or indirectly in the manifestation of ME/CFS, owing to their crucial roles in cell signaling and energy metabolism as well as existing clinical research. Further, a good place to start looking for potential biomarkers that could link LDIR exposures and ME/CFS is the mitochondrion, especially when considering the demonstrable involvement of mitochondrial enzymes in RIBEs [92,147,153,154,155,156]. An example of a mitochondrial protein that may fit this characterization of multifariousness is ORNT1, which is encoded by the gene *SLC25A15* and is involved in numerous cellular processes known to be implicated in both ME/CFS and RIBEs.

### 2.5. SLC25A15 Overview and Role in Cellular Metabolism

Mitochondrial ornithine transporter 1 (ORNT1) is a protein that is essential for the urea cycle [157]. The urea cycle is a primary liver metabolic pathway that converts ammonia, which is produced during protein metabolism, into urea destined for excretion. The mitochondrial catabolism of glutamine follows this pathway and produces ammonia and α-ketoglutarate, which is shunted to the TCA cycle [158]. Ammonia is converted to carbamoyl phosphate in mitochondria and then is used with ornithine to produce citrulline, which is then transported outside mitochondria for further processing in the cytosolic branch of the urea cycle. Ornithine is transported into the mitochondrial matrix via ORNT1 and is utilized in both arginine biosynthesis and the detoxification of ammonia [157,159]. In the context of disease, hyperammonemia can cause lethargy, difficulty concentrating, confusion, neurotrauma and even death in severe cases [158]. The broad symptomatology of urea-cycle disorders has some overlap with ME/CFS, including lethargy, malaise, nausea, and cognitive issues; however, these symptoms do not typically emerge during a period of recovery following physical exertion and are primarily linked to protein intake. There is research that suggests that the urea cycle may be implicated in some ME/CFS cases [23,24,123,160].

It is currently unknown whether ORNT1 plays a direct role in the pathogenesis of ME/CFS, and there is a dearth of research on the subject. One large-scale study of ME/CFS patients identified that *SLC25A15* sequence variants may play a role in the disease [161,162], although further research is required to confirm this finding. While *SLC25A15* has otherwise not been directly investigated within ME/CFS etiology, mutations in *SLC25A15* have been identified in ornithine translocase deficiency or hyperornithinemia-hyperammonemia–homocitrullinuria (HHH) syndrome, and there is comparatively considerable research on the etiology of this disease and the involvement of ORNT1 [163]. HHH is a rare autosomal recessive condition that is effectively an inborn error of the urea cycle; because specific mutations can cause instability and malformation of ORNT1, mitochondria in liver cells cannot effectively uptake ornithine for participation in the mitochondrial branch of the urea cycle, which causes buildup of ammonia from protein metabolism [164]. Treatment of the disorder is largely based on management of symptoms and may require the administration of drugs that sequester ammonia and nutritional supplements that provide arginine [165]. The primary symptoms of the disease include extreme tiredness in infants and episodic symptoms in adults, which are likely mediated by ammonia accumulation in the blood and consequent neurotoxicity [166].

The role of *SLC25A15* in HHH may serve as a potential case study for how aberrant mitochondrial function and cascading biological effects can contribute to disease. It has been suggested that secondary mitochondrial dysfunction may develop over time in HHH [167,168]. This concept coincides with clinical findings showing “large and bizarre mitochondria in liver, muscle, leukocytes and fibroblasts of some HHH patients” [168]. The same study showed that HHH patients had elevated liver enzymes, lactic acidemia, increased urinary excretion of lactate, and a low pyruvate relative to lactic acid. These findings are consistent with broader mitochondrial dysfunction in HHH [169,170], which may be the result of aberrant mitochondrial metabolism compensating for reduced cellular energy production; because impaired transport of ornithine for use in the mitochondrion occurs, this deficiency could disrupt related pathways which are reliant on a normal metabolic flux to function properly [166,171]. Specific pathways that are immediately affected involve amino acid metabolism and nitrogen balance; however, over time this could potentially lead to aberrant metabolism in related pathways to compensate for reduced substrate and energy availability. The urea cycle is linked to the metabolism of alanine and aspartate as well through the activity of arginosuccinate synthetase (ASS) [172,173]. ASS facilitates the synthesis of arginosuccinate from citrulline and aspartate [172]. A downstream enzyme in the urea cycle, arginosuccinate lysase (ASL), catalyzes the breakdown of arginosuccinate to fumarate and L-arginine [174,175]. Fumarate participates in the TCA cycle and is also produced by the dehydrogenation of succinate by mitochondrial respiratory Complex II [176,177]. These connections are metabolic clues that may point towards fatigue symptoms in HHH being linked to aberrant energy metabolism and reduced cellular energy efficiency, provided that further research is conducted in this area to confirm these clinical findings. Both ASS and ASL functional mutations are also included under urea cycle disorders and share aspects of symptoms with HHH [170,174].

A combination of factors is likely to cause fatigue symptoms in the context of HHH. A dysfunctional urea cycle most immediately causes accumulation of ammonia, neurotoxicity, and lethargy symptoms [23,178,179]. Secondary mitochondrial dysfunction due to compensatory shifts in metabolism to maintain energy and metabolic homeostasis may result in lower ATP production, potentially causing reduced energy availability. Finally, this metabolic imbalance may cause problems in linked metabolic pathways, such as the TCA cycle [24,125], pyruvate metabolism [131], amino acid synthesis [180], and oxidative phosphorylation [138,139], which may in turn result in energy insufficiency. In terms of neurological effects, a combination of these factors could account for cognitive difficulties, malaise, and fatigue symptoms.

In contrast to the extensive evidence in the HHH literature, there is some research that indicates that ME/CFS patients may have abnormal levels of ammonia in their blood post-exertion [21]; however, the research is relatively sparse. As was previously covered, hyperammonemia has been linked to fatigue symptoms [181]. Moreover, other groups have noted deficiencies in urea cycle metabolism in some ME/CFS patients [182], although further research is required to confirm any candidate biomarkers.

A few older clinical studies found decreases in glutamine and ornithine concentrations in the blood plasma of ME/CFS patients [183,184]. These studies further suggested that altered energy metabolism may be present in some ME/CFS patients, specifically in the TCA cycle and function of pyruvate dehydrogenase [143]. As previously outlined [4], impaired metabolism was reported and is suspected to cause compensatory upregulation of mitochondrial Complexes I and V, enzymes in upstream pathways (e.g., TCA cycle), amino acid catabolism, and fatty acid catabolism in the mononuclear cells of ME/CFS patients. In cerebrospinal fluid, one recent clinical study [180] found elevation of trans-aconitate, which is involved in the TCA cycle. The same study found elevation of serine and changes in lipid metabolism compared to healthy controls. Other clinical studies have found increased lactate in the ventricles of ME/CFS patients [185,186,187,188], possibly indicating dysfunction of normal oxidative phosphorylation in these tissues, pending additional experimental confirmation. It would be very interesting to further explore these aspects of altered metabolism in ME/CFS patients in a clinical context and investigate how such alterations may contribute to disease emergence and severity. Again, there seems to be considerable evidence that links mitochondrial involvement in ME/CFS in one form or another [125]. Characterizing the function of ORNT1, ASS, ASL, and other associated proteins in ME/CFS patient samples from various tissues (e.g., cerebral–spinal fluid, SMBCs, skeletal muscle, etc.) could shed further light on the etiology of the disease or even indirectly elucidate dysfunction in different nodes of the metabolic network within the context of ME/CFS. Furthermore, investigating these enzymes in patient samples could be used to further metabolically profile ME/CFS patients, eventually leading to the possible segregation of ME/CFS patient groups into distinct disease subtypes that can be understood effectively on a mechanistic level and clinically managed.

### 2.6. SLC25A15 in LDIR Exposure

*SLC25A15* has not been extensively investigated in the context of LDIR exposure; *SLC25A15* is noted to be upregulated in some cancers, including bladder carcinomas, gastric cancer, and melanoma [189]. Increased *SLC25A15* expression in one study [190] was associated with a significant inhibition of apoptosis and progression of prostate cancer. It has been noted that mitochondrial amino acid transporters can effectively reprogram the metabolism of cancer cells by regulating the flux and creation of anabolites crucial for rapidly proliferating tumor cells [189]. We have previously reviewed low-dose radiation exposure in the context of ME/CFS [191] and proposed a putative model based on overlapping biomarkers that integrates possible RIBEs with ROS generation, potential downstream metabolic and immunologic effects relevant to ME/CFS, and possible hematopoietic dysregulation that may further be connected to melanoma in the context of ME/CFS. Additionally, we conducted a broader review of the possible association with ME/CFS and cancer in an earlier report [192].

Mechanistically, *SLC25A15* is integrated in the same pathways pertinent to low-dose radiation-induced bystander effects. RIBEs have long been known to involve modulation of cellular metabolism [93,94,153,155,193,194]. RIBEs have been demonstrated to cause calcium fluxes [89,195,196], reduced mitochondrial metabolism [92,154,194,197], and programmed cell death in various experimental contexts [198,199,200,201,202]. For example, a recent study using an ex vivo model found that incubation of cells in irradiated tumor-conditioned medium found a significant reduction in mitochondrial OXPHOS, ATP production, glycolysis, and leucine secretion [193]. It was also found that photonic bystander signals [150,203,204] directly inhibited the activities of Complexes I and V and inhibited ATP production in cultured human epithelial cells, which is a finding that coincides with other RIBE research evidencing the involvement of mitochondrial metabolism in mediating the effect. Further research points to the role of reactive oxygen species (ROSs) and oxidative stress in RIBEs, which can be generated by mitochondria as a byproduct of OXPHOS.

It is possible that *SLC25A15* could be directly modulated by bystander signals or even participate in the cellular responses to and propagation of the signal given its crucial role in cellular metabolism. There is currently a lack of studies in the literature that indicate the direct involvement of ORNT1 in RIBEs. It is clear that ORNT1 and other mitochondrial transporters could play a very important role in the mitochondrial aspects of RIBEs, given the extensive literature strongly supporting mitochondrial involvement in cellular responses to LDIR and signaling. This is a possible avenue for future research in ME/CFS and RIBEs that could uncover its importance in mediating or participating in both. It is likely that, if such research were undertaken, a complex picture of metabolic modulation in the context of the disease could become evident that would likely elucidate its etiology further and possibly point to areas of overlap with RIBEs and associated effects.

Because both RIBEs and ME/CFS represent complex biological processes, it is almost certain that, in a whole organism, any observed adapted energy metabolism is not limited to specific tissues or processes such as detoxification of ammonia in the liver. It is also evident that a key area of research in ME/CFS involves the role of immunity in the manifestation of the disease, along with crosstalk between immune and metabolic pathways. Another protein that bridges metabolic functions with immunity that has also been identified in ME/CFS literature is the CD38 glycoprotein.

### 2.7. CD38 Overview and Possible Connections to ME/CFS

CD38 is a membrane-associated protein found on the surface of many types of immune cells, including monocytes, dendritic cells, granulocytes, B-lymphocytes, and NK cells [205]. CD38 has two primary known functions: a transmembrane receptor involved in immunity, and an enzyme assisting in NAD+ catabolism and regulation of intra- and extracellular NAD+ concentration [205,206,207,208,209]. As a transmembrane receptor, CD38 can complex with and activate T cells, promoting cytokine production and release [205]. CD38 is also involved in the regulation of B-cell activation in antigen recognition [205]. As an enzyme, CD38 catalyzes the conversion of NAD+ into ADP ribose (ADPR) and cyclic ADP ribose, which is believed to serve a crucial role in regulating intracellular NAD+ concentration [207,210]. Additionally, ADPR is known to regulate calcium ion flux as it binds to and activates TRPM2 [209,211]. NAD+ and its reduced form NADH are well known in their role as electron carriers and oxidizing agents in glycolysis, pyruvate oxidation, the TCA cycle, oxidative phosphorylation, beta phosphorylation, and other catabolic pathways [126,127,212].

Several studies have shown alterations in immune surface cell receptors in ME/CFS patients [213,214], and ME/CFS has long been suspected to have an immunological component [215]. One paper using in vitro techniques examined the metabolic profile of B cells from ME/CFS patients and found lower mitochondrial mass, significant usage of essential amino acids, and significantly increased expression of CD38 following stimulation compared to healthy control samples [135]. The authors concluded that a stress response was likely triggered in ME/CFS B cells, resulting in increased usage of substrates for ATP production. Another PBMC study [121] found evidence of downregulated interferon signaling and expression of immunoglobulin genes, suggesting immune suppression in the cohort of ME/CFS patients used for the study. Another clinical study [213] found significantly higher expression of both CD38 and ion channel genes crucial for calcium signaling in NK cells from ME/CFS patients; specifically, TRPM2. Further, baseline cytotoxicity was significantly reduced in these ME/CFS patients. These findings suggest possible compensatory activity in ME/CFS in an attempt to ameliorate dysregulation of calcium ion homeostasis and to promote restoration of NK cytotoxicity, although further research is required to substantiate this hypothesis. Another research group found that severe ME/CFS was associated with higher circulating B-lymphocytes along with higher pro-inflammatory cytokines such as IFN-gamma, TNF, and IL-17 [56]. Another clinical study [216] observed significant changes in B-cell subtypes, T cells, and other biomarkers, suggesting “significant impairments in immune regulation in ME/CFS”. Some studies have also tentatively linked these effects to possible dysregulated metabolism in ME/CFS [25] due to CD38’s dual function as a surface receptor and regulator of NAD+ within and outside the cell. We discuss these findings more extensively in a previous review that provides possible connections to diseases such as melanoma [191].

Overall, increased CD38 expression in various types of immune cells in ME/CFS patients reported in a number of clinical studies suggests that perhaps persistent immune stimulation and inflammation may be a common factor in the disease. There appears to be a broad appreciation in the literature now that immune involvement in most ME/CFS cases is probable [217]. An interplay between immunity and metabolism is further suspected given the detection of metabolic abnormalities in the B-cells of ME/CFS patients [135]. A potential link between immunity and metabolism can be investigated by interrogating CD38’s role as an enzyme and as a key regulator of NAD+ metabolism. Increased CD38 activity in the context of inflammation or immune activation likely drives depletion of NAD+ [218], which is required for normal cellular metabolism. It is suspected, though not yet confirmed, that depletion of NAD+ can also disrupt mitochondrial function in ME/CFS [219]. Indeed, reduced NAD+ is associated with decreased oxidative phosphorylation (and ATP synthesis), a possible greater reliance on glycolysis, compensatory shifts in metabolism, and ultimately dysfunction of immune cells [207,220,221]. However, this putative cascade has yet to be extensively investigated in the context of ME/CFS.

In a recent clinical pilot study [222], a select cohort of ME/CFS patients were administered the anti-CD38 antibody daratumumab. The authors found that six patients of ten experienced considerable improvements in their symptoms, with four patients experiencing no significant clinical changes. They demonstrated that a reduction in serum IgG levels was associated with self-reported clinical improvement, while low baseline NK-cell counts were associated with a lack of clinical response to the drug. These findings are a positive development in the testing of effective treatments for some ME/CFS cases, but further research is required to determine efficacy across patient cohorts and larger sample sizes [223]. Moreover, further research is required to determine whether altering CD38-related immune pathways could benefit at least a subset of ME/CFS patients in broader clinical practice.

### 2.8. CD38 in RIBE

CD38 has not yet been extensively investigated for potential involvement in RIBEs. Further, there is overall limited current evidence for the role of LDIR and RIBEs in altering organism immunity [224]. This is likely because a majority of such immunological studies focus on dose ranges pertinent to tumor radiotherapy, where higher doses of radiation are typically employed for killing tumor cells. Due to comparatively less focus on low-dose effects, where RIBEs are observed as the predominant biological effect in most experimental contexts, there is very little research on the interplay between the immune system and RIBEs. Much research has been conducted in vitro on bone marrow stem cells and cells of related lineage that were indispensable in first demonstrating and further elucidating RIBEs and radiation-induced genomic instability (RIGE), among other related LDIR effects. It is likely, given the effects observed in in vitro models, that immune signaling is modulated by RIBEs and other LDIR effects, even if our current understanding of RIBEs in radiation and immune therapy responses is incomplete [225]. Multiple groups have observed varied effects of RIBEs both in vitro and in vivo models, which include modulation of cellular metabolism [93,94,193,226,227], the modulation of mitochondrial metabolism [92,154,155,193,194], the production of reactive oxygen species [89,95,98,154,155,156,194,228], exosome signaling [146,147,148,149,150,151], calcium fluxes [89,195,196], the induction of apoptosis [198,199,200,228], cell-cycle arrest [226,229], and the release of secondary signals that perpetuate responses in neighboring, LDIR-unexposed cells.

These mechanistic observations and evidence from in vitro and in vivo studies warrant a further investigation of multifunctional proteins such as CD38 in the context of LDIR responses and disease. CD38, as previously described, is intimately involved in both immune regulation and in controlling levels of intra- and extracellular NAD+, which is a crucial electron carrier involved in virtually all aspects of normal cellular metabolism. In addition, CD38 catalyzes the formation of products that activate calcium channels (i.e., TRPM2 via ADPR), which are crucial in normal calcium signaling. The loss of CD38 function has been clinically associated with impaired immune responses, dysfunctional metabolism, and impaired mitochondrial function [230,231]. It would be very interesting to further probe the role of CD38 in RIBEs and determine how its function modulates NAD+ availability, mitochondrial health, and calcium fluxes to potentially facilitate observed effects.

It is known that many different enzymes and metabolites coordinate to maintain metabolic homeostasis under normal conditions. Another potential candidate biomarker of interest that integrates mitochondrial metabolism, immune function, and the cell cycle is cyclin D1.

### 2.9. Cyclin D1 Overview and Possible Role in ME/CFS

Cyclin D1 (*CCND1*) is a protein that regulates the cell cycle via complexation with specific cyclin-dependent kinases (CDK4 or CDK6) [232]. Complexed cyclin D1 regulates the transition from G1 to S phase by inactivating tumor suppressor proteins (such as pRb, p21) via phosphorylation or sequestration and allowing E2F transcription factors to transcribe S phase genes [232]. In cancers, the overexpression of cyclin D1 correlates with tumor progression and protection from apoptosis. Due to genomic instability present in most cancers, the amplification or translocation of oncogenes (including *CCND1*) and consequent overexpression is a common biomarker for prognosis [233]. In B-cell lymphoma, for example, *CCND1* is usually translocated upstream of the IgH promoter, and similar chromosomal translocations are observed in some cases of multiple myeloma [233,234].

Cyclin D1 also binds to several nuclear receptors independently of CDK4/6 and thereby modulates hormonal signaling, cell growth, proliferation, and differentiation [235]. Increased cyclin D1 is observed in cells during the G1/S phase transition due to signaling in both Ras-driven mitogenic pathways and other pathways [236,237,238]. There is also some evidence of cyclin D1 in epigenetic transcriptional regulation; cyclin D1 has been observed binding to histone acetylases and deacetylases, which regulate the expression of genes involved in differentiation and cell proliferation [235]. It is, therefore, more appropriate to describe cyclin D1 as a regulator of the cell cycle, transcription, histone code, and metabolism, and as a signaling molecule sensitive to numerous growth factors, cytokines, and stress signals [235,239].

The multifunctional role of cyclin D1 is relevant to discuss here because some of the cell types examined in ME/CFS research do not actively divide (e.g., skeletal muscle fibers). In terms of potential causes of fatigue-related symptoms and immune dysfunction, the possible involvement of cyclin D1 in ME/CFS can be linked to mitochondrial pathways. Research has shown that aberrant cyclin D1 expression can cause mitochondrial dysfunction [240], the suppression of mitochondrial OXPHOS [241], altered immune cell function [242], and increased cellular stress signaling [243]. This is likely due to the complex role of cyclin D1 (-CDK4/6) in regulating cellular growth and metabolism. Cyclin D1-CDK4/6 can phosphorylate and inactivate PGC-1α, which is a transcriptional coactivator crucial for mitochondrial biogenesis and oxidative metabolism. Conversely, the overexpression of PGC-1α can increase cyclin D1 levels since PGC-1α increases ATP production but can also work to decrease oxidative stress through the expression of genes involved in free-radical scavenging, such as superoxide dismutase (SOD) [244,245,246]. The inhibition of PGC-1α may result in reduced numbers of mitochondria, decreased respiratory capacity, and the impaired production of ATP [247,248,249,250,251]. As such, PGC-1α has been identified as a protein of interest in ME/CFS research [138,252,253], although much work must be done to determine whether an aberrant function of PGC-1α is present in most ME/CFS patients and whether aberrant cyclin D1 activity plays a role in any observed effect since there is little primary research on ME/CFS patients and models currently.

Cyclin D1 is known to widely reprogram cellular metabolism for the G1/S phase transition. The expression of cyclin D1 in this context promotes glycolysis, reduced OXPHOS, and more shunting of metabolites to anabolic pathways such as the pentose phosphate pathway. As described previously, several studies have demonstrated similar metabolic shifts in the immune and muscle cells of ME/CFS patients [136,252,254,255,256], although none so far have proposed a link to potentially aberrant cyclin D1 activity.

Several studies have indicated that ME/CFS patients appear to have chronic, low-grade immune activation, and many others have demonstrated the involvement of the immune system in the disease [10,22,26,28,118,120,121,131,135,138,139,217]. The expression of cyclin D1 can be mediated by the activation of IL-6 and TNF-α signaling, which also activates mitogenic growth pathways [257]. Additional research is required to establish a connection to cyclin D1 activity in the context of ME/CFS. Oxidative stress or the dysregulation of redox homeostasis, however, has been directly implicated in ME/CFS in a few studies [5,28]. In theory, with chronic low-grade immune activation, cyclin D1 expression can also be sustained, which could possibly drive the effects observed in ME/CFS studies, but there does not appear to be a study currently linking abnormal cyclin D1 expression or signaling to ME/CFS, and much additional preclinical and clinical research is required to substantiate this hypothesis. As with ORNT1 and CD38, the integrated and multifunctional role that cyclin D1 plays in cellular metabolism, the cell cycle, and the signaling pathways suggests that it may be involved in ME/CFS in at least a subset of patients.

### 2.10. Cyclin D1 and Connections to LDIR Effects

Cyclin D1 has been investigated considerably in the context of high-dose radiation effects due to its role in several cancers and potential as a target in cancer radiotherapy [234,237,238,245]. However, many studies have demonstrated that cellular regulation of cyclin D1 within radiation responses is highly dependent on dosage, with differential regulation observed at doses within the RIBE range. An interesting study [258] found that cyclin D1 is degraded in tumor cells following a high radiation dose of 10 Gy, whereas it was actually stabilized when the same human tumor cells were exposed to a 500 mGy fractionated dose over the course of several weeks. The authors of this study also discussed the finding that cyclin D1 overexpression occurs as a radiation-induced adaptive response (RIAR) in the context of adaptation to fractionated doses, and that overexpression of cyclin D1 also results in genomic instability in the irradiated cells. The authors further deduce that repression of cyclin D1 expression is “likely to cancel the harmful effects of long-term exposure to [fractionated radiation doses]”, and therefore, cyclin D1 may be used as a potential biomarker of long-term exposure to radiation [258]. Another study [259] found similar bimodal cyclin D1 regulation in human keratinocytes that depended on dosage; cyclin D1 was found to be induced by LDIR (100 mGy X-radiation) and localized to the cytoplasm, while higher doses (5 Gy gamma-radiation) resulted in nuclear accumulation. The authors also concluded that, likely through direct association with the pro-apoptotic Bax protein, cyclin D1 can improve inner mitochondrial membrane potential in cells exposed to low doses and thereby promotes an anti-apoptotic response in stressed cells [259]. As a crucial regulator of the cell cycle, apoptotic signaling, and cellular metabolism, these observations make sense because low doses of ionizing radiation are known to affect cells differently than high doses [260,261,262,263,264,265,266].

In the context of RIBEs and other LDIR research, these findings of the bimodal regulation of cyclin D1 are not surprising. Many LDIR studies have reported, for decades now, that cellular responses are highly context-dependent and are essentially disparate when compared to high-dose effects where the primary deleterious biological effects stem from direct or indirect DNA damage and responses (repair, programmed death, etc.) to this damage. These data are also further evidence that the LNT model, which still informs much of radiation protection and practical medical concepts of radiation exposure on health, cannot be used to adequately capture the complexity of cellular or organismal effects below the low-dose threshold. While this is obviously not direct evidence that low doses of radiation could potentially be a stressor associated with ME/CFS pathogenesis and/or maintenance of pathophysiological processes, it is abundantly clear that our current understanding of low-dose radiation effects, RIARs, and RIBEs must be enhanced with additional research to preclude or maybe even eventually confirm this possibility. Given the existing research pointing to similar syndromes in populations exposed to low doses [62,63,106] and commonalities in observed biological effects between low-dose RIBEs and ME/CFS, including altered cellular and mitochondrial metabolism [93,94,125,138,139,155,193,194], the regulation of mitochondrial membrane potential [131,138,154,155,267], the induction of cellular stress pathways [28,118,153,197,267], the generation of reactive oxygen species [86,87,93,143,268,269,270,271,272], the emergent albeit significant observed effects on specific OXPHOS enzymes (e.g., mitochondrial respiratory complexes) [124,128,132,136,138,154,155,267], the involvement of cytokine and exosome signaling [140,146,147,148,150], and other processes covered in this review, we believe that further investigation is warranted in this area and may prove highly fruitful in furthering our understanding of LDIR effects and how these effects may possibly be implicated in ME/CFS with additional research.

### 2.11. Alpha-Synuclein Overview and Possible Connections to ME/CFS

Potentially bridging putative neurological effects, alpha-synuclein (α-syn) is a neuronal, membrane-associated protein that is believed to be closely involved in the regulation of synaptic vesicles. This effect has been observed primarily in dopaminergic neurons [273]. Though its role is not completely understood, in healthy neuronal tissue, α-syn localizes to presynaptic terminals and mediates the mobility and clustering of synaptic vesicle pools [274]. Through this activity, α-syn is believed to play a buffering role in neurotransmitter release. It also plays a role in SNARE complex assembly and stabilization, where it is believed to function similarly to a chaperone protein by ensuring the efficiency of the machinery that facilitates vesicle fusion under conditions of repeated neuronal firing [275,276]. A-synuclein has also been demonstrated to modulate dopamine synthesis and functions to limit the concentration of cytosolic dopamine in presynaptic boutons; this is because high concentrations can result in the generation of ROSs and other toxic metabolites and cytokines through monoamine oxidase (MAO) activity [277,278]. MAO is bound to the outer membranes of mitochondria in most human tissues [279,280]. Further, high concentrations of dopamine can cause the formation of highly reactive dopamine quinones (DAQs), either enzymatically or via auto-oxidation. Some of these byproducts of elevated dopamine concentrations and dopamine catabolism, especially 3,4-dihydroxyphenylacetaldehyde (DOPAL) generated by MAO and in close proximity to mitochondria, can result in the induction of mitochondrial membrane permeability, the attenuation of mitochondrial Complex I and respiration, and the loss of inner membrane potential [277,279,280,281,282,283,284,285,286]. In turn, these effects may promote apoptotic signaling, the increased generation of ROS, further oxidative insult, and the pathological aggregation of α-syn in neurodegenerative disease, specifically PD [280,281,283,284,285,286]. It is conceivable that these effects could generate a positive feedback loop of ROS generation and stress signaling that may cause the degradation of dopaminergic neurons and contribute to or cause PD [284,285,286], although these mechanisms are still under investigation in the PD literature today.

There are a few additional areas of research into the normal and pathological function of α-syn that are still emerging and are, as of now, less established. Some research suggests that α-syn could be involved in vesicle transport between the endoplasmic reticulum and the Golgi body, as well as mitochondrial dynamics, which likely involve membrane interactions [276,287]. Additionally, some research suggests that defective α-syn can potentially translocate to the nucleus and may indirectly prevent the shuttling of key proteins involved in transcriptional regulation, such as DNMT3A [288,289,290,291]. However, these effects are still under investigation, and their relevance to disease is unclear.

There is currently no strong evidence that dysfunctional α-syn is present in ME/CFS. There is nevertheless potential in researching the possible dysregulation of α-syn in ME/CFS; because it sits at an interesting position linking neuronal signaling, membrane interactions, mitochondrial biology, and redox stress, identifying patterns of expression in patient cohorts could help isolate subgroups where neuronal symptoms remain unexplained. Additionally, due to its sensitivity to redox stress, α-syn function could be a candidate biomarker for redox imbalance assays in ME/CFS if future work identifies deleterious α-syn function in ME/CFS patients.

Currently, no studies have established this connection. One particular case report published several years ago concerning an autopsy of a patient who had died with a prior diagnosis of ME/CFS found several neuropathological abnormalities, including a loss of white matter and several axonic aberrations [292]. The study further described considerable tissue insult in white and gray matter and prominent amyloid deposits with neurofibrillary tangles present in the frontal cortex, basal ganglia, and thalamus. Though the authors did not identify the involvement of α-syn directly, they argued that these findings warrant further studies into CNS diseases that may be associated with ME/CFS or ostensible presentation of ME/CFS.

Mitochondrial dysfunction could again possibly serve as a mechanistic link. A-syn localizes to the outer mitochondrial membrane, especially under conditions of cellular stress [293,294]. The overexpression or misfolding of α-syn has been shown to impair Complex I activity and ATP production [293,294,295]. Oxidative stress has been demonstrated to promote α-syn misfolding and appears to be exacerbated by it as well. As previously reviewed, many ME/CFS studies report reduced mitochondrial efficiency [128,132,152], aberrant mitochondrial function including oxidative phosphorylation [124,125,136,254,296,297], and redox imbalances [123,129,143,255,268,270,271,298,299]. It could be that these processes somehow promote subclinical α-syn misfolding, and while not producing the same aggregation threshold seen in PD, the alteration of conformation or localization of α-syn may occur in some cases and could possibly contribute to observed neuroinflammatory effects. Further research is needed to demonstrate that this could be a mechanism in some ME/CFS cases, as research in the area is sparse.

A-syn also plays a role in neuroinflammation and innate immune response. The protein is expressed in immune cells, including microglia and monocytes. In vitro, picomolar concentrations of α-syn aggregates were demonstrated to sensitize toll-like receptor 4 (TLR4) in a model of PD [300]. Many studies have investigated this pathway in the context of PD [300,301,302,303] and furthered the idea that misfolded α-syn may act as a damage-associated molecular pattern (DAMP), leading to the triggering of immune responses in PD. It is interesting to note that low-grade neuroinflammation and innate immune activation appear to be present in most ME/CFS patients, according to several clinical studies [17,25,119,120,215,216,304]. It is possible that very low levels of α-syn aggregates could promote neuroinflammation in ME/CFS, although further research is required to justify this hypothesis experimentally.

Without additional research, α-syn remains a possible biomarker that could be relevant to ME/CFS. There exist no postmortem studies examining α-syn expression or conformation that have demonstrated evidence of involvement to our knowledge. Further, no studies of CSF or plasma samples from ME/CFS patients exist that have examined α-syn expression or conformation. In PD patients, some studies have shown an association between oligomeric α-syn in CSF and plasma and the severity of fatigue symptoms [305,306]. We propose overall that more research in this area could provide useful information on the disease mechanisms associated with ME/CFS and whether aberrant α-syn activity is relevant to the manifestation of fatigue symptoms.

### 2.12. Alpha-Synuclein and Radiation Exposure

In radiation therapy, the upregulation of α-syn is associated with fatigue symptoms [307]. Idiopathic PD is believed to involve many different environmental factors, including ionizing radiation exposure; possible LDIR mechanisms are still under investigation [308,309]. One study even found that low-energy EM radiation can promote α-syn aggregation, oxidative stress, and cytotoxic effects in human neuroblastoma cells [310], although further research is required to confirm these findings.

It is possible that low-dose radiation exposures could affect the monomer-to-oligomer regulatory mechanisms in cells and thereby contribute to abnormal function, although much additional research is required to substantiate this idea.

## 3. Recommendations for Future Research

**Figure 2:**
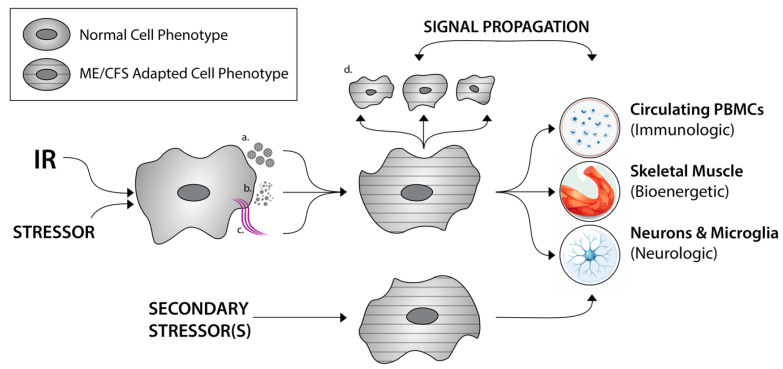
A representation of how a stressor or set of stressors affecting one tissue could possibly give rise to diverse effects in distant tissues via signal propagation. An initial stressor, such as ionizing radiation (IR), is known to be capable of inducing the secretion of exosomes (a), soluble factors (b), and UV biophotons (c) from cells in the context of RIBEs. Exposure to these stressors could promote further secondary signaling in bystander cells (d), as has been reported in the literature and discussed in this review. The communication of these stress signals could, in theory, produce effects in distant tissues through circulating factors or even promote maintenance of the diseased state in response to additional cellular stress events. This model has yet to be confirmed in the etiology of ME/CFS; however, we hypothesize that these effects could possibly promote establishment of an “ME/CFS Adapted Cell Phenotype” in patients that promotes emergence of symptoms and disease progression.

ME/CFS is a complex disease that likely involves many aspects of cellular metabolism and mitochondrial dynamics, immune and inflammatory processes, and neurological mechanisms that, together, contribute to a diseased state. Further, because no universal set of accepted criteria for ME/CFS diagnosis exists, patient groups in studies are likely to be heterogeneous in presentation. Here, we propose future research directions that could assist in efforts to determine how the reviewed biomarkers could potentially be involved in ME/CFS and how stressors such as LDIR exposure could possibly be implicated in ME/CFS through these mechanisms. Figure 2 illustrates the hypothesis that LDIR exposure could induce diverse cellular responses in distant tissues through RIBE signal propagation.

### 3.1. Putative Biomarkers of Interest in ME/CFS and RIBE Research

Firstly, diagnostic criteria must be standardized across studies. A core set of diagnostic criteria must first be adopted to ensure that patients with representative “canonical” symptoms (i.e., post-exertional malaise) can be assessed. The Canadian Consensus Criteria or the IOM Criteria should be employed and strictly adhered to due to their relatively conservative criteria [8]. However, to capture ME/CFS patients that may not fit standard diagnostic criteria but still fit the broad symptomatology of ME/CFS, we believe an additional group should be included to capture atypical presentations if comorbidities are excluded [8,9,311]. While this group may not present all the typical symptoms and possibly present varied pathophysiologies and biomarker profiles, evaluating similar biomarkers between the groups could still potentially reveal common or divergent areas of underlying dysfunction. The establishment of a secure, shared database open to ME/CFS researchers could prove invaluable in future studies. A positive development over the past few years has been the founding of a UK ME/CFS biobank [162,312], which aggregates ME/CFS specimens along with comprehensive patient health profiles, which has already led to considerable research interest and the analysis of samples [313,314,315,316,317]. Research efforts such as these establish the foundations required for enabling full cross-study meta-analyses on ME/CFS.

Alongside this database, the recruitment of large prospective cohorts of individuals with detailed histories of radiation exposure should also be performed. As previously reviewed, these would include nuclear workers, medical radiology patients, Chernobyl and Fukushima survivors, and atomic veterans. A comprehensive data collection would include documenting the extent of exposure, comprehensive health histories, including any comorbidities, and any existing symptoms or history of symptoms that overlap ME/CFS. This would further allow the stratification of participants into subgroups that correlate with ME/CFS subtypes.

### 3.2. Integrating Transcriptomics, Proteomics, Metabolomics, and Epigenomics

Considerable sample collection would need to be undertaken for these studies to cover various biomarkers and tissues with suspected relevance to ME/CFS. The establishment of a repository of whole blood samples (for PBMCs, other biomarkers), skeletal muscle biopsies, CSF, urine samples, and others would facilitate further research and metabolic profiling of patients. A focus on PBMCs and skeletal muscle would involve cellular bioenergetics biomarkers with an emphasis on quantifying mitochondrial function. Additionally, data on ORNT1 activity, CD38 expression and activity, cyclin D1 localization, and α-syn regulation and oligomerization should be collected and analyzed against healthy controls. Targeted assays for lactate/pyruvate ratios, TCA intermediates, amino acids (orthinine, arginine), NAD+/NADH, the urea cycle, and oxidative stress markers should also be conducted to establish detailed metabolic profiles of patients. In blood and other samples, evaluation of DNA methylation patterns in PBMCs and non-coding RNAs present in circulating exosomes could be useful in determining whether circulating DAMPs modulate these effects, which seems to have been suggested by previous studies [140].

### 3.3. Exosome Profiling

The characterization of exosome content can serve as a method to identify ME/CFS and RIBE biomarkers and mitochondrial dysfunction, as well as the propagation of damage signals. The quantification of exosomal mtDNA, miRNA, lncRNA, and protein cargo (e.g., ROS, α-syn, inflammatory cytokines) could be conducted in plasma before and after controlled exercise tests. These results could be compared with exposure to bystander signals or low-dose radiation in vitro and with samples from radiation-exposed cohorts to uncover any potential overlap. Exosome profiles can then be correlated with symptom severity, post-exertional malaise, and the presence of additional circulating inflammatory cytokines to further build out this model. Furthermore, the in vitro testing of ICCM and serum from ME/CFS patients using cultured reporter cells could help elucidate whether signaling cascades in RIBEs and ME/CFS share overlap or if any disparities exist.

### 3.4. Functional Mitochondrial and Calcium Assays in Patient-Derived Cells

The collection of PBMCs and B-lymphocytes from ME/CFS patients and radiation-exposed cohorts could prove useful in determining functional mitochondrial impairments and correlation with fatigue symptoms. Additional endpoints can be incorporated to analyze the role of calcium fluxes or whether aberrant signaling is present in these samples. The measurement of basal and maximum respiration, ATP production, and proton leak can be conducted in real time using extracellular flux assays (such as Seahorse XF) [318,319]. Mitochondrial potential can be measured using the JC-1 fluorescence assay among other assays for the verification of apoptotic signaling via loss of inner mitochondrial membrane potential [320,321,322]. ROS generation can be measured via several methods, such as live dichlorofluorescein diacetate (DCFDA) fluorescence microscopy or using MitoSOX [323,324,325]. Further, fluorescence-based calcium flux assays using stains such as Fura-2 AM [326,327] can be pursued to determine whether any correlation exists between this signaling and mitochondrial function in patient groups. These assays should also be repeated in vitro using radiation- and bystander signal-exposed cells to confirm any potential findings. Moreover, the supplementation of NAD+ precursors (such as nicotinamide riboside, nicotinamide mononucleotide) or PGC-1α agonists should be tested for potential rescue of mitochondrial function if any impairments are detected.

### 3.5. Mechanistic Studies of RIBEs on Immune–Metabolic Crosstalk

In order to further connect low-dose effects to immune function, an additional in vitro investigation is required. The use of a co-culture system of irradiated immortalized cells and PBMCs can be used to investigate this connection. The generation of bystander signals upon irradiation can be observed by assessing the release of exosomes, ROSs, and cytokines from exposed cells [96,146,147,148,149,153]. Further assays on bystander cells, including metabolic effects (i.e., NAD+ levels), mitochondrial respiration, and inflammatory responses, can then be conducted before and after manipulation of CD38 activity to assess whether such manipulation promotes or attenuates bystander effects. The attenuation of CD38 activity can be accomplished by small molecule inhibitors such as daratumumab [222]. The additional characterization of ORNT1 expression and activity in irradiated cells or knockdown of *SLC25A15* could be used to further link these effects to mitochondrial function. These models can be further improved by incorporating animal models of fatigue [188] and investigating the role of LDIR exposure in the modulation of fatigue symptoms and biomarker profiles.

### 3.6. Mechanistic Studies of RIBEs on Cyclin D1 Activity

The apparent bimodal activity of cyclin D1 between high and low radiation doses warrants further investigation in the context of RIBEs [258,259]. The expression and localization of cyclin D1 can be evaluated in PBMCs from radiation-exposed and non-exposed groups. These results can be compared to PBMC samples from ME/CFS groups to assess whether there are any similarities or significant differences in the regulatory activity of cyclin D1 upon low-dose radiation exposure.

### 3.7. Further Exploration of α-Synuclein as a Neuroinflammatory Biomarker

Previous studies have shown that incredibly minute quantities of α-syn aggregates can sensitize microglia via TLR4 and TLR2 and promote neuroinflammatory responses [300,302,303]. The measurement of CSF and plasma oligomeric α-syn should be conducted in all patient cohorts; this can then be correlated with neurocognitive scores, sleep quality, and fatigue severity. This could help elucidate whether oligomeric α-syn is potentially involved in these symptoms and whether a dose threshold exists for manifestation of these effects. Further research can be conducted in vitro using neuronal cell lines such as T98G to determine whether LDIR exposure or bystander signaling can produce a similar effect. Of course, these studies could include additional metabolomic and transcriptomic characterization to potentially connect these effects to other pathways suspected in ME/CFS pathogenesis.

### 3.8. Linking Clinical Phenotypes to Molecular Subtypes

Once considerable preliminary data has been collected, a computational analysis could be conducted to segregate clinical phenotypes and associated biomarkers. This could be achieved by machine learning processes, such as unsupervised clustering of multi-omics data to identify molecular subgroups within ME/CFS, as previous studies have attempted [19,116]. For example, categories could include “immune-driven”, “mitochondrial deficit-driven”, and/or “neuroinflammatory” subgroups and groups that overlap these categories. These clusters can be validated against clinical metrics employed in ME/CFS, such as the duration of post-exertional malaise, exercise intolerance, and neurocognitive deficits. These efforts could help develop a panel of biomarker tests for ME/CFS and effectively cluster patients into different subtypes, which could then inform further research and eventually clinical management options based on the underlying observed abnormalities in metabolism, immunity, and neurocognition.

### 3.9. Development and Testing of Targeted Interventions

Following the clustering of molecular subtypes of ME/CFS, the clinical testing of various therapies can proceed. Further pilot trials of anti-CD38 therapy in stratified subgroups could be promising in some cases [135,222]; in these studies, the monitoring of immune phenotypes, cytokine profiles, and mitochondrial function should be performed as well to determine any modulatory effects. The modulation of metabolism in some cases of ME/CFS could ameliorate fatigue and associated symptoms. The supplementation of diet with NAD+ precursors and antioxidant compounds could be employed to determine whether this provides benefit to any patients; however, more prospective research is needed to determine whether such efforts are likely to be effective in most patients.

### 3.10. Creation of a Centralized ME/CFS-Radiation Exposure Registry

With the establishment of an ME/CFS database and considerable historical data on individuals exposed to ionizing radiation, longitudinal studies could be conducted on radiation dose (incorporating personal dosimetry, medical imaging records), infection history, lifestyle factors, symptoms, and clinical outcomes for patients. This would enable real-time epidemiological analyses to detect any dose-responsive relationships with ME/CFS-like symptom clusters.

### 3.11. Additional Research Recommendations

It is clear that research into the causes of ME/CFS and underlying molecular mechanisms can only be successful if a truly interdisciplinary approach is used. Such efforts would involve coordinating many different researchers and clinicians from a variety of subdisciplines. The formation of a consortium combining clinicians, molecular biologists, radiation physicists, epidemiologists, bioinformaticians, and patient advocacy groups would assist in streamlining future research efforts. The ability to share raw datasets via secure platforms would also be an asset to strengthen future findings. Further, the publishing of and adherence to various standard operating procedures for assays and other procedures would help ensure reproducibility between groups.

Eventually, we envision the use of emerging evidence to refine guidelines on low-dose radiation safety (e.g., occupational limits) and on screening for fatigue syndromes in radiation-exposed populations. The incorporation of routine assessment of immune–metabolic biomarkers into clinical care pathways for patients presenting with unexplained chronic fatigue would be beneficial.

## 4. A Hypothetical Model for the Induction and Maintenance of ME/CFS upon Low-Dose Radiation Exposure

In this next section, we propose a potential model for how ME/CFS pathogenesis could possibly emerge as a result of research into the various putative biomarkers covered in this review. This model is hypothetical but serves to tie together the various players and processes relevant to ME/CFS which are associated with emerging research. It should be noted that much additional research is required to substantiate this model; however, we hope that discussing the potential links between these pathways could assist future research in connecting crosstalk between these pathways and identifying other studies in the literature that have investigated similar effects.

### 4.1. Exposure to Stressors and Potential Downstream Effects

**Figure 3:**
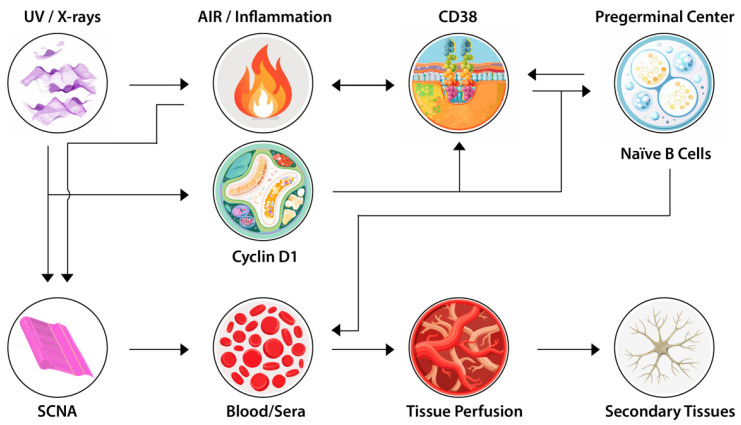
A diagram of a possible model for emergence of ME/CFS upon exposure to ionizing radiation. Exposure to IR triggers an exaggerated stress response in proximal cells, which induces a broader inflammatory response and, in turn, modulates CD38 activity in affected cells. Aberrant α-syn aggregation may be mediated directly by IR or possibly a secondary effect of inflammatory processes or Abnormal Immune Response (AIR). Circulating damage-associated molecular patterns (DAMPs; such as α-syn) may convey this stress response to distant tissues. Further research is required to confirm this signaling cascade and its possible relevance to the health effects of low-dose radiation exposure and ME/CFS.

An initial stressor promotes one or more dysfunctional metabolic or signaling processes in affected tissues. This could be the result of an infection [26,32,34], the persistence of an inflammatory state from a previous infection [118,119,129], or radiation exposure [62,63,104,105,106]. It could also be that a combination of stressors can contribute to the initiation of the pathological state. This stressor or set of stressors that can contribute, though infection and others have been linked, is still unknown. For example, ionizing radiation exposure could promote the modulation of adaptive responses at low doses [259,328,329,330], which are mediated by the immune system [96,331,332,333,334,335]. This could lead to an adaptive or radioprotective response in exposed tissues or bystander cells receiving signals from directly exposed cells. This process could, for example, involve the promotion of CD38 expression or maintenance of CD38 expression, which has been linked to radioresistance in cancer cells [206,207,210,218,220,231]; increased CD38 activity or expression has been observed in ME/CFS patients as well in some studies [135,222]. This expression could possibly drive the depletion of NAD+, leading to downstream modulation of cellular metabolism and mitochondrial effects in tissues directly affected by the stressor. CD38 upregulation drives the downstream modulation of cellular metabolism and mitochondrial effects via depletion of NAD+, which is, as covered previously, required as an oxidizing agent in glycolysis, pyruvate oxidation, the TCA cycle, oxidative phosphorylation, beta phosphorylation, and other catabolic pathways [126,127,212,230]. Figure 3 shows a potential model for these effects that could, in theory, facilitate localized tissue damage, modulation of inflammation and immunity, neurological effects, and metabolic alterations in ME/CFS. 

In terms of specific effects observed in ME/CFS patients, and by way of example, B-cell abnormalities have been noted in many ME/CFS studies [216,336,337,338,339,340,341]. Pregerminal (naïve) B cells often express CD38; however, this expression gradually decreases as they mature into memory or antibody-secreting cells [342,343,344,345]. Exposure to low doses of ionizing radiation or bystander signal could promote radioresistance [346,347,348] yet ultimately compromise the metabolic homeostasis of these cells [135,230]. Other proteins may be affected in response to a stressor or set of stressors, such as cyclin D1 in LDIR responses [258,259]. The downstream dysregulation of OXPHOS and CD38 activity [208,218,220,221,231] in neuronal tissues may lead to minute quantities of alpha-synuclein oligomers, which act as DAMPs that further promote a neuroinflammatory state [275,279,280,284,285,286,294,295,300,349].

From this initial tissue insult by the stressor or stressors, downstream effects can ensue, and signal propagation may continue to pass stress responses to other areas of the body [56,115,122,140,179,304]. This may also be perpetuated upon exposure to additional stressors following the initial exposure, or repeated exposures to the same stressor. For example, the secretion of bystander factors (e.g., exosomes) from cells exposed to bystander factors (signal propagation) or directly irradiated cells communicates ROS [97,98,272], cytokines [96,335], miRNAs [146,149], mtDNA [147], other damage signals to secondary bystander cells [150,198,203,204,350,351], and potentially ones far away from the site of initial exposure; signal propagation continues. Immune dysregulation and the receipt of inflammatory signals drive the upregulation of CD38 in response, which drives mitochondrial dysfunction due to a depletion in NAD+ in various tissues. Oxidative phosphorylation is also potentially affected, along with other metabolic pathways. Compensatory shifts in metabolism occur to cover deficiencies in energy and substrate production.

The upregulation of CD38 also occurs in immune responses, which could account for higher expression observed in ME/CFS patients and the theme of persistent low-level immune activation in ME/CFS [17,25,119,135,205,208,214,215,216,218,304]. Increased consumption of NAD+ due to dysregulation of CD38 also results in increased synthesis of ADPR [207,210], which activates TRPM2, a mitochondrial channel crucial for calcium signaling and apoptotic responses [209,211]. It should be noted that TRPM2 is highly expressed in the central nervous system [352,353,354], which could partly account for pathological neurological effects if further investigated and confirmed experimentally [355,356,357]. In response to oxidative stress across various tissues (due to metabolic modulation and stress signaling), additional proteins are activated, such as PARP, which also generates TRPM2 agonists (intracellular ADP-ribose) [352,355,356,357]. Additional pathways are affected by this dysregulation, and this could also affect further degradation of redox homeostasis. The dysregulation of metabolism promotes a dysregulated immune response with evidence of persistent low-level activation. Immune responses may be additionally triggered via subsequent or concurrent stressors, such as viral infection. CD38 mediates inflammatory responses in innate immune cells like neutrophils, monocytes, and macrophages, helping control infections by influencing their recruitment and function at infection sites [135,205,208,218,343,344]. The altered metabolism and triggering of immune responses may result in energy depletion and a “vicious cycle” of low-grade immune activation, persistence of adaptive response to the stressor via signal propagation, and depletion of substrates and energy used for normal metabolism.

Long-term, low-dose exposure to a stressor (such as radiation) can promote the dysregulation of cyclin D1 in affected tissues [258,259]. Cyclin D1 expression in repeated LDIR exposure is stabilized, and this promotes a RIAR [258,259]. Cyclin D1 localizes to the nucleus and coordinates with BAX to inhibit apoptotic signaling [358,359,360,361,362]. This may occur in PBMCs in response to damage signals, which might also entail the metabolic reprogramming of these cells that could affect their function [216,337,338,339,340,341,363]. The upregulation of CD38 as a RIAR to LDIR, damage responses, and other stressors may also occur. The dysregulation of OXPHOS may promote highly toxic alpha-synuclein oligomerization in neuronal tissue, which, while not at the levels of clinical PD, promotes inflammatory responses as extracellular DAMPs in neuronal tissues [275,279,280,284,285,286,294,295,300,349]. The intracellular modulation of mitochondrial metabolism may also occur via membrane associations of alpha-synuclein [279,280,284,285,293,294]. The stabilization of cyclin D1 as a stress-adaptive response promotes cellular metabolic reprogramming and modulates cellular growth, proliferation, and differentiation in various affected tissues [258,259]. Cyclin D1 may promote the shunting of metabolites to anabolic pathways and away from catabolic, ATP-generating pathways, contributing to the persistence of the dysregulated state of metabolism [244,245]. PGC-1α may be phosphorylated and inactivated by complexed cyclin D1 to further promote these effects [249,250,251]. Further, the epigenetic regulation and reprogramming of gene expression via histone code may occur via cyclin D1 activity [235] to further entrench the altered metabolism and, consequently, the diseased state.

### 4.2. Signal Cascade

Damage responses may initially be relatively muted and considered subclinical in ME/CFS. Genetic predisposition or synergistic effects due to a combination of stressors may promote the emergence of clinical disease [161,313,316,364]. The assortment and order of these stressors, or the number of repeated instances of a single kind of stressor, required to produce clinical disease are likely highly variable between individuals. However, the emergence of a concerted hypometabolic and neurocognitive response occurs when some unknown threshold is met. This threshold is likely highly variable because of comorbidities, lifestyle, or genetic factors. This could also partially account for the heterogeneity of biomarker profiles in ME/CFS patients in many studies [15,256,365].

Damage signal propagation leads to effects all over the body via circulating exosomes (which carry various factors) [140], inflammatory cytokines [122,140,304,366,367], PBMCs that may produce secondary signals, and potentially other factors, such as alpha-synuclein acting as a DAMP. Broad cellular metabolic reprogramming and inflammation ensue initially as an adaptive response to a stressor but become persistent due to the further generation of signals. Symptoms manifest due to a combination of metabolic and inflammatory/immune responses affecting neuronal and musculoskeletal tissues and the persistence of an adaptive response. Secondary effects of dysregulation can occur in other tissues, accounting for associated symptoms in ME/CFS and observations of irregularities in the metabolites of some ME/CFS samples.

### 4.3. Possible Clinical Manifestations

Under this hypothetical model, several deductions could be made if these mechanisms are confirmed by experimental research. If confirmed, these findings would assist by providing specific targets for therapy that could depend on the metabolic and immune phenotype of the patient being treated. PEM in this model is likely to be the result of a combination of factors, including dysregulated metabolism and impaired mitochondrial function in select tissues, and an exaggerated adaptive response to stressors. Unrefreshing sleep can also be explained by dysregulated metabolism and neuroinflammation, which can lead to a vicious cycle of not getting enough restful sleep and this causing worsening symptoms due to the addition of a new stressor. Muscle/joint pain is likely to be the result of dysregulated metabolism and aberrant increases in lactic acid generation due to the shunting of metabolites away from more energetically favored and oxygen-dependent pathways. Headaches and dizziness could also be the result of neuroinflammation and dysregulated metabolism. These effects must be further investigated in the literature, as noted above, before any definitive conclusions can be made.

### 4.4. Model Limitations

While this model represents a possible means by which an ME/CFS phenotype could emerge upon LDIR exposure, it remains largely hypothetical at present due to a lack of replicated, high-quality data in the literature. There are, therefore, considerable limitations with this model in its current state. As previously discussed, this model should not be perceived as comprehensive and should be viewed as complementary to existing infectious, autoimmune, and neuroinflammatory models. This model also does not explain or expand on differences in signaling between cells and tissues, as this is still under active investigation in ME/CFS research [116,120,135]. Further, this model does not address the mechanisms by which genetic background can influence disease severity for the same reasons [161,313]. For example, it is evident from the literature that RIBEs are highly variable, depending on factors such as cell type radiosensitivity and genetic background [272,350,368]. Further, the identification of appropriate cohorts of ME/CFS patients may be problematic, as previously discussed, because of inconsistent screening criteria and the lack of an established biomarker (or set of biomarkers) that may be used to segregate patient groups based on potential molecular subtypes [7,13,160].

The major limitations in identifying targets for future investigation include the lack of high-quality, clinical research that would better inform this model with respect to potential targets and mechanisms. The identified candidates for further research have not been tested extensively in the context of ME/CFS and by multiple research groups. ORNT1 dysfunction and *SLC25A15* sequence variants have been directly identified in ME/CFS patient samples [161,162], but these findings must be repeated and confirmed by additional studies. Currently, ORNT1 is suspected to be involved in ME/CFS primarily based on the presence of sequence variants in ME/CFS patients, as well as potential mechanistic connections to mitochondrial function and energy metabolism. ORNT1 does not appear in current RIBE or LDIR research but has been interrogated under higher doses and is embedded in well-characterized RIBE signaling pathways [92,153,168,189,194,267]. CD38 overexpression has been directly observed in NK cells from ME/CFS patients [135,213], but these findings must be repeated and confirmed by additional groups before any consensus is achieved. The results from these papers and other studies indicate possible aberrant calcium signaling in ME/CFS. CD38 is suspected primarily due to the finding of overexpression and aberrant function in ME/CFS PBMCs and possible mechanistic links to downstream cytokine release and immune function [56,121,135,213,216]. CD38 has not been identified in RIBE or LDIR research; however, a mechanistic link is suspected due to its role mediating calcium fluxes that have been well characterized in RIBEs [195,196,208,218,220]. Cyclin D1 dysfunction and mutations have not been identified in the current ME/CFS literature. The suspected altered function in some cells and tissues primarily stems from potential mechanistic links to immune function [232,242,245,359], oxidative stress responses [243], and inflammatory responses [235,245,360], which may be mediated by cyclin D1 in relevant pathways involving the cell cycle [360,369], the modulation of cellular metabolism [244,245], mitogenic pathways [236], and cell differentiation [232,258,370]. Pathways involving cyclin D1 and cell-cycle checkpoints are suspected in ME/CFS based on mechanistic links; however, no ME/CFS study has explicitly examined cyclin D1 as of today. Therefore, much additional research is required to support the hypothesis that aberrant cyclin D1 activity contributes to ME/CFS. Cyclin D1 has been implicated in RIBE cell-cycle checkpoint effects in vitro [229,258,259,369]. Alpha-synuclein dysfunction and mutations have not been identified in ME/CFS in any large-scale study. The suspected involvement in ME/CFS relies partly on a case report of an autopsy of a patient previously diagnosed with ME/CFS [292] and more strongly by its mechanistic links to mitochondrial function [284,285,286,349], deleterious activity under oxidative stress [281,286,349], and a potential role in neuroinflammation [275,288,290,293] as well as its potential role as a DAMP in other diseases [275,291,300,303]. Alpha-synuclein has not yet been identified in LDIR or RIBE research. We have provided a qualitative summary of the available research in Table 1.

Overall, there is great uncertainty concerning whether the possible biomarkers covered in this review are directly implicated in ME/CFS and RIBEs because these candidate biomarkers have not been extensively researched in the context of ME/CFS and RIBEs separately. Nevertheless, it appears likely, given the evidence from the literature covered in this review, that a broader examination of the cellular and extracellular processes mediated by these proteins would be fruitful in understanding the etiology of ME/CFS and possibly demonstrate overlap or differences with LDIR responses (such as RIBEs). To address these limitations, the scope of biomarkers covered in this review should be expanded in future research, and high-throughput approaches may be necessary to collect high-quality data from patient cohorts. Pertinent cell signaling pathways (e.g., mitogenic, pro-inflammatory, etc.) and key components in these pathways should be examined closely in future studies, and variance in expression/function between different tissues (e.g., PBMCs vs. skeletal muscle vs. CSF) would also have to be undertaken to obtain a more complete picture of the pathophysiological processes underpinning the disease.

Finally, the model does not directly address the complexities and nuances of immune responses in ME/CFS, pro-inflammatory cytokine signaling, and low-grade inflammation. Researchers are actively investigating the possibility of using anti-cytokine therapy in ME/CFS in limited trials, yet these therapeutics are under active investigation and have not been approved for broader use [371,372,373,374,375,376]. Nevertheless, many cytokine pathways are currently under investigation for potential targets. For example, some researchers have investigated the JAK/STAT signaling pathway and are testing JAK inhibitors [375,376,377]. The inhibition of JAK in lymphocytes prevents cytokine (such as interferons, interleukins, etc.) signal transduction, which suppresses pro-inflammatory signaling. Currently, the results from these studies have not yielded consistent and widespread symptom remission across patient cohorts but are still ongoing.

## 5. Conclusions

In summary, the convergence of recent evidence from studies encompassing LDIR biology and ME/CFS biomarkers points to LDIR as a plausible, yet still greatly underexplored, stressor that could potentially initiate or amplify the pathogenic cascade underlying ME/CFS. The reviewed biomarkers, including mitochondrial respiratory impairment, CD38-driven NAD+ depletion, cyclin D1-mediated metabolic reprogramming, and α-synuclein–induced neuroinflammation, highlight common pathways where environmental conditions might intersect with individual susceptibility to produce the heterogeneous clinical spectrum of chronic fatigue and associated symptoms within the context of LDIR exposure. As reviewed, while these pathways are suspected to be potentially involved in ME/CFS, there is currently little to no direct experimental or clinical evidence that links LIDR exposure to ME/CFS pathogenesis. Future investigations should prioritize large, high-quality cohorts and combine rigorous diagnostic criteria with standardized multi-omics profiling, exosome analysis, and functional mitochondrial assays in order to confirm the largely unexplored and otherwise tenuous links reviewed in this report. We hope that future research will facilitate the identification and testing of targeted interventions, possibly including but not limited to CD38 inhibition and NAD+ supplementation, to first determine applicability and eventually manage or even treat ME/CFS once suitable targets have been identified. By integrating these approaches, we can potentially promote the elucidation of mechanisms underlying ME/CFS in at least a subgroup of patients whose disease can be linked to LDIR exposure and related biological effects.

## Figures and Tables

**Table 1:** A qualitative summary of the quality of the available literature on the various putative biomarkers covered in this review. Categories include the availability of direct ME/CFS research in vitro, clinical ME/CFS studies, RIBE research in vitro, and RIBE research in vivo (mostly animal models). Scores represent a qualitative measure of data availability: o = very few articles in the literature; x = some limited evidence in the literature with several articles; xx = considerable evidence in the literature with many studies but no broad consensus; and xxx = overwhelming evidence in the literature and established (or burgeoning) acceptance.

	ME/CFS; In Vitro	ME/CFS; Clinical	RIBE; In Vitro	RIBE; In Vivo
Mitochondria	xx	xx	xxx	xxx
ORNT1	o	x	o	o
CD38	x	xx	o	o
Cyclin D1	o	o	xxx	xx
Alpha-syn	o	o	o	o

## Data Availability

No new data were created or analyzed in this study. Data sharing is not applicable to this article.

## Abbreviations

The following abbreviations are used in this manuscript:

ME/CFS	Myalgic Encephalomyelitis/Chronic Fatigue Syndrome
CFIDS	Chronic Fatigue and Immune Dysfunction Syndrome
CCC	Canadian Consensus Criteria
LDIR	low-dose ionizing radiation
RIBE	radiation-induced bystander effect
IOM	Institute of Medicine Criteria
PEM	post-exertional malaise
ICF	idiopathic chronic fatigue
PAIS	Post-Acute Infection Syndrome
PRS	Post-Radiation Syndrome
LNT	Linear no-threshold (model)
mSv	millisievert
EBRT	external beam radiation therapy
OXPHOS	oxidative phosphorylation
ATP	adenosine triphosphate
PBMC	peripheral blood mononuclear cell
TCA	tricarboxylic acid
PCS	Post-COVID Syndrome
mtDNA	mitochondrial DNA
ICCM	irradiated cell-conditioned medium
HHH	hyperornithinemia-hyperammonemia-homocitrullinuria
ASS	arginosuccinate synthetase
ASL	arginosuccinate lysase
TRPM2	transient receptor potential cation channel subfamily M member 2
ADPR	ADP-ribosylation
PD	Parkinson’s Disease
Gy	Gray (J/kg)
